# Changes in Oscillatory Brain Networks after Lexical Tone Training

**DOI:** 10.3390/brainsci3020757

**Published:** 2013-05-03

**Authors:** Edith Kaan, Ratree Wayland, Andreas Keil

**Affiliations:** 1 Department of Linguistics, University of Florida, P.O. Box 115454, Gainesville, FL 32611, USA; E-Mail: ratree@ufl.edu; 2 Department of Psychology, University of Florida, P.O. Box 112766, Gainesville, FL 32611, USA; E-Mail: akeil@ufl.edu; 3 Center for the Study of Emotion & Attention, University of Florida, P.O. Box 112766, Gainesville, FL 32611, USA

**Keywords:** gamma, alpha, phase synchrony, speech perception, learning, lexical tones

## Abstract

Learning foreign speech contrasts involves creating new representations of sound categories in memory. This formation of new memory representations is likely to involve changes in neural networks as reflected by oscillatory brain activity. To explore this, we conducted time-frequency analyses of electro-encephalography (EEG) data recorded in a passive auditory oddball paradigm using Thai language tones. We compared native speakers of English (a non-tone language) and native speakers of Mandarin Chinese (a tone language), before and after a two-day laboratory training. Native English speakers showed a larger gamma-band power and stronger alpha-band synchrony across EEG channels than the native Chinese speakers, especially after training. This is compatible with the view that forming new speech categories on the basis of unfamiliar perceptual dimensions involves stronger gamma activity and more coherent activity in alpha-band networks than forming new categories on the basis of familiar dimensions.

## 1. Introduction

### 1.1. Lexical Tones

Languages use different combinations of acoustic and phonetic features to form speech categories. When learning a foreign language, one needs to learn which features and/or combinations of features are relevant in order to form representations of these new speech categories in memory. The ease with which a learner can do this depends on the degree and type of correspondence between the foreign language and native language categories [1,2,3,4]. The learning of speech categories therefore provides an excellent case to study changes in neural networks associated with category learning. The present study analyzes oscillatory brain activity (gamma and alpha band) to investigate changes in neural networks associated with the learning of Thai lexical tones by native speakers of languages that either have or do not have tonal distinctions. 

Lexical tones, characteristic of tone languages such as Mandarin Chinese, Thai, and Yoruba, are differences and changes in voice pitch (F_0_) used to distinguish among word meanings. For instance the Thai syllable [k^h^a:] means “galangal root” when pronounced with a low-falling tone; “leg” when spoken with a low-falling-rising tone; “I, servant” when it has a high-falling tone; “to do business in” when it has a high-rising tone; and “to be lodged in” with a mid-level tone. To perceive differences between tone categories, the listener needs to combine various perceptual cues such as the height of the onset pitch, the pitch slope, the timing of turning point of the pitch slope, the height of the offset pitch, as well as the duration of the syllable, amplitude and voice quality [5]. Tone languages differ in the number of tone categories, and in what combination and values of features constitute a category. In general, having a tone language as a native language will make it easier to perceive, identify and categorize foreign linguistic tones [6,7,8,9,10,11,12,13]. This has been attributed, in part, to differences in the weighing of perceptual cues: whereas speakers of non-tone languages, such as English, are sensitive to differences in onset pitch, average and offset pitch, speakers of contour-tone languages, such as Mandarin Chinese, are more sensitive to curvilinear pitch contour, e.g., [14,15,16,17].

Training can improve the processing of lexical tones by non-tone language speakers, mainly by inducing shifts in the perceptual weighing of features, e.g., [8,18,19,20]. For instance, a study comparing native speakers of English learning Mandarin Chinese with naïve native English speakers, found that the learners’ experience with Chinese had shifted their perceptual focus from the average pitch to the pitch slope [18]. Training can also induce shifts in neural activation. After a short laboratory training on identifying tones in Mandarin Chinese, English speakers showed an increase in activation in brain areas that were active pre-training (left superior temporal gyrus), but also recruited additional areas, such as the right inferior frontal gyrus [21]. In a related study, better learners showed an increase in left-hemispheric areas after training, whereas poorer learners showed an increase in right-hemispheric areas [22]. These results suggest that neural networks engaged in the processing of foreign speech categories change with training and proficiency. 

Effects of both short-term training and native language background on tone perception have been investigated by Kaan and colleagues [12,23]. Kaan *et al.* [12] tested native speakers of English, Mandarin Chinese and Thai in an event-related brain potential (ERP) oddball study before and after a two-day laboratory training on Thai lexical tones. A Mismatch Negativity (MMN) was found for deviant *versus* standard Thai tones in all groups. This component was, however, largest in the English group before training, especially for low-falling tones. After training, the MMN in the English group decreased to the level of the other groups. Since the low-falling tone deviants in the study differed in onset pitch from the mid-level tones used as standards, these data support the view that the English speakers were initially more sensitive to these onset pitch differences, but that they changed their perceptual focus to other pitch dimensions after a short-term categorization training. This interpretation was supported by behavioral data: even though the native English speakers showed the largest MMN, this group performed worse on a tone discrimination task before training compared with Thai and Chinese groups. This is expected if the English speakers did not focus on pitch contour. After training, however, the English group performed at the level of the Thai and Chinese on the discrimination task, suggesting that they changed their perceptual focus. 

### 1.2. Oscillatory Brain Activation

The Kaan *et al.* [12] study, and many other studies investigating the neural mechanisms underlying the acquisition of foreign speech categories, employed event-related brain potentials (ERPs), or their magneto-encephalography (MEG) equivalents, e.g., [24,25,26,27]. In these studies, the averaged electro-encephalographic signal EEG or MEG is analyzed, time-locked to the onset of a stimulus. EEG oscillations need not be phase-locked to the stimulus, however. Hence, some changes in activation may be obscured by time-locked averaging. In the present study, we investigated changes in EEG oscillations in the frequency domain to lexical tones, before and after training.

In the present study, we focused on changes in the gamma (typically 30–120 Hz) and alpha (typically 8–13 Hz) frequency range. Gamma-band power, and phase synchrony in this frequency band, have been hypothesized to reflect the integration of activation in different neural areas, and, hence, the combination of different perceptual features [28]. Enhanced gamma-band activity has been associated with selective attention to features [29,30,31,32], perceptual encoding in memory [33,34], maintenance in working memory [35], and the mapping of a stimulus onto representations in long-term memory, e.g., visual object representations [32,36], lexical-semantic representations of words [37,38], and environmental sounds [39]. Power in the gamma band has been shown to increase as a function of learning, supporting the idea that gamma-band activity is involved also in the formation of new combinations of features. For instance, increases in power and coherence in the gamma band have been observed in classic conditioning paradigms, in which a light of a particular color was associated with an electric shock [40]; or the presentation of particular speech sounds was associated with aversive noise [41]. These findings suggest that gamma-band power increases during the learning of new associations. Since the learning of foreign language tone distinctions involves the combination or re-combination of different pitch dimensions, we were interested in seeing whether the learning of foreign tone categories was associated with changes in the gamma frequency band.

Learning does not always implicate an increase of gamma-band activity, however. In a recent study, participants were trained for five days in a visual search paradigm [42]. Power in the gamma band first increased and then decreased over the course of training. Power in the gamma band correlated negatively with power in the alpha band (8–14 Hz), the latter showing a U-shaped function. The interpretation of alpha-band activity is still controversial, see [43,44]. One interpretation is that an increase in alpha-band activity reflects active inhibition of areas that are not relevant to the task [43]. However, increases in alpha-band activity have also been reported over task- relevant areas and may therefore also index active processing [44]. Another interpretation is that alpha-band activity reflects the ease of processing. More difficult tasks have generally been associated with a decrease of alpha-band activity, suggesting that alpha-band activity is inversely related to processing efficiency [45,46]. Regardless of the interpretation of alpha-band activity, Hamame *et al.* [42] distinguish two phases of learning: one in which the number and/or strength of the neural connections increase to form a new neural representation (increase in gamma-band activity, decrease in alpha-band activity); and a second phase in which coding becomes more efficient by restricting the representation to the strongest or most selective connections, as indicated by a decrease in gamma-band activation and an increase in alpha-band activity [47]. 

Motivated by the Hamame *et al.* study [42], we focused on changes in gamma and alpha-band activity during the processing of foreign tones as a function of language background and training. We compared learners, who were initially unfamiliar with the tones used, before and after a training to see if gamma-band activity increased and alpha-band activity decreased as a result of training, especially in learners whose native language did not use pitch to distinguish among words.

In addition to changes in spectral power, one can study the phase synchrony between electrode sites of a particular EEG frequency band. Phase synchrony (coherence) in the gamma band has been hypothesized to underlie the formation of neural, multi-featural representations [40], with novices engaging different neural networks than more experienced learners. With respect to language learning, coherence in the gamma band (30–40 Hz) has been found to be stronger over the right hemisphere in native speakers of German who were low-proficient in English, compared with those who were high-proficient in English, especially when listening to English rather than their native language [48]. As for changes in alpha-band synchrony, low-proficient German speakers of English showed a stronger alpha-band coherence when attending to radio and TV reports in English compared with a non-linguistic control task, especially over the left hemisphere. Highly proficient speakers of English showed such an increase in coherence for the language tasks only over left-hemispheric temporal sites, and showed a reduction of coherence over prefrontal electrodes. These findings suggest that low-proficient second-language speakers recruit a wider and more coherent neural network than highly proficient speakers. Similar differences in the alpha band between the two proficiency groups were found when they were listening to their native language, however. It is therefore unclear to what extent the effects observed are due to long-term exposure to a second language, or to general language exposure and/or aptitude [49].

### 1.3. The Current Study

In the current study we explored changes in the gamma and alpha frequency bands to the processing of foreign speech (lexical tone) contrasts; in particular we were interested in seeing to what extent gamma and alpha-band activity was affected by the native language background of the participants as well as short-term experience (laboratory training). We analyzed the EEG data previously collected in a passive oddball study by Kaan *et al.* [12], and investigated changes in mean spectral power and in phase synchrony between electrode sites for high-rising and low-falling Thai tones presented as standards or deviants. Analyzing the overall differences in oscillatory activation between the groups, as well as the difference between standards and deviants would allow us to see to what extent results from the time-frequency analysis would correspond to results from the ERP analysis reported by Kaan *et al.* [12]. In this study, native speakers of Thai, English and Mandarin Chinese were tested in the odd-ball paradigm before and after a two-day discrimination training on the Thai tones. Since each participant served as his or her own control, we could test the effect of short-term experience avoiding the between-group confound in [49]. In particular, we were interested in the following comparisons. (1) *Differences between the language groups before training*. Because the stimuli used were Thai words pronounced with Thai tones, we expected the native Thai participants in the study to be different from the Chinese and English speakers in alpha and gamma-band activity. In addition, since Mandarin Chinese speakers were already familiar with certain tone dimensions through their native language, whereas English speakers were not, we were interested in pre-training differences between these two groups in terms of alpha or gamma-band power. In the ERP analysis reported by Kaan *et al.* [12], the largest difference between the English group compared to the other groups was found in the comparison between deviant and standard low-falling tone conditions before training. We were therefore interested in seeing whether a similar result pattern could be observed in the time-frequency analysis. Finally, since previous studies reported difference in coherence for different language proficiency groups [48,49], we were interested in differences in phase synchrony between the native English and Chinese groups before training. (2) *Differences in the effect of training between the native English and the native Chinese language groups*. The English group, not being familiar with lexical tones and having to shift their perceptual focus from onset pitch to pitch contour [14,16,18], was expected to differ from the Chinese group in the strength of the gamma- and alpha-band power as a result of training. Based on Hamame *et al.* [42], the English group was expected to show a larger increase in gamma-band power and a larger decrease in alpha-band power as a result of training compared with the Chinese group. In addition, we were interested in seeing whether training would affect the phase synchrony in the alpha and gamma-bands differently in the native English compared with the native Chinese group. 

In terms of the topographical location of synchrony and power differences, this study was mainly exploratory in nature, and we did not have any strong predictions regarding the spatial distribution of the oscillatory differences expected.

## 2. Experimental Section

### 2.1. Participants

Analysis was conducted on data collected in a previous study [12]. Written informed consent was obtained from all participants, according to procedures approved by the University of Florida Institutional Review Board. The original data included 12 native speakers of English, 12 native speakers of Mandarin Chinese, and 11 of Thai. None of the English speakers had any experience with a tone language, and none of the Mandarin Chinese speaking participants had any experience with a tone language other than their native tongue. We selected data from 10 native speakers of American English (3 women, 7 men, mean age 22.0), 10 native speakers of Mandarin Chinese (5 women, 5 men, mean age 27.2) and 11 native speakers of Thai (6 women, 5 men, mean age 27.7). Data from these participants were selected on the basis of having at least 50 artifact-free trials per condition. On average, 24%, 18% and 26% of the data were rejected for the Chinese, English and Thai group, respectively.

Five of the English participants, two of the Chinese, and three of the Thai reported to have some musical experience. Musical experience has been shown to affect the perception and acquisition of lexical tones, e.g., [19,50,51,52]. Musical experience, however, did not have any effect on the effects reported in the main text for the alpha-band power and phase synchrony measures. Power in the gamma band was stronger for musicians than non-musicians at lateral sites (*F*(1,27) = 4.58, *p* < 0.05). Effects involving language reported for the gamma-band power in section 3 remained significant when the participants with musical experience were removed from analysis (*F*(2,16) = 21.40, *p* < 0.001).

### 2.2. Stimuli and Procedures

Stimuli and procedure have been described in detail in Kaan *et al.* [12]. Stimuli were nine spoken tokens of the syllable [k^h^a:], synthesized on the basis of one naturally spoken instance of that syllable, pronounced with a mid-level tone by a female native speaker of Thai. This token was shortened to 450 ms. The pitch contour was then manually changed to yield a Thai low-falling and high-rising tone. Next, two additional tokens of the mid-level, high-rising and low-falling tones were generated by shifting the entire F0 contour −15 Hz and −30 Hz. All stimuli were normalized for peak amplitude (98% of the scale). The nine stimuli thus obtained were presented to two native Thai speakers (one male and one female) and were judged to be acceptable exemplars of the intended tone categories.

In the EEG study, participants watched a silent movie while stimuli were presented over headphones. Four blocks were presented, with the order of the blocks randomized between participants: one in which the mid-level tone was presented as standard with the low-falling tokens as deviants (10%; or 120 deviant tokens among 1080 standards); one in which the low-falling tokens were presented as standards, with the mid-level tone as deviants; and two blocks that were similar to the two mentioned except that the high-rising tones, rather than the low-falling tones served as deviants and standards, respectively. Participants answered comprehension questions about the movie after each block. EEG was recorded from 39 Ag/AgCl scalp electrodes at a sampling rate of 512 Hz, referenced to the left mastoid and arithmetically re-referenced to the averaged mastoids. 

The EEG study was conducted before and after a two-day identification training of 1 h (162 stimuli) per day. In this training participants heard one token at a time and were trained to categorize the tones as category A (low-falling), B (mid-level) or C (high-rising) [9,10]. All three participant groups underwent training. However, since the syllabi were real words in Thai, the comparison between the Thai and the other groups before training should be interpreted with caution. In addition, undergoing a training of well-known tone categories and words may involve different processes and have different effects than training of unknown categories, or syllables that do not have meaning. Since the effect of training may be hard to interpret for the Thai, we focused primarily on comparing the effect of training for the English and Chinese participants.

### 2.3. EEG Analysis

EEG data were analyzed in the following way. First, artifact-free epochs of −300 to 700 ms relative to the onset of the stimulus were identified and subjected to wavelet analysis of the single trials. Time-frequency representations of this signal were established using convolution of the signal with complex Morlet wavelets as described in detail elsewhere [32,53]. In the present study, complex Morlet wavelets *g* were generated in the time domain for different analysis frequencies *f*_0_:

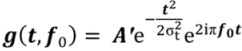
(1)
with *A′* depending on the parameter *σ_f_*, specifying the width of the wavelet in the frequency domain, the analysis frequency *f*_0_ and the user-selected ratio *m*:

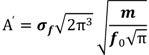
(2)
with

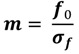
(3)


A constant *m* = *f*_0_/σ_f_ equal to 9 was selected to achieve good time and frequency resolution in the frequency range of interest, which was 8 to 80 Hz in our study, at a resolution of 1 Hz. Wavelets were normalized to have equal amounts of energy and were then applied to the single-trial signal at each electrode. For each artifact-free epoch, time-varying energy in a given frequency band was obtained as the squared absolute value of the convolution of the cosine-square-tapered EEG signal with the wavelet. Single trial time-by-frequency matrices were then averaged in order to obtain the evolutionary spectrum for each electrode, and experimental condition, in a given participant. An epoch from 280 to 100 ms prior to stimulus onset was used as an estimate of general noise. The mean of this baseline epoch was subtracted from the time-by-frequency matrices for each frequency and time point for each electrode, respectively.

In addition to time-varying spectral power, we analyzed the inter-site phase-locking of the neural oscillations measured with the wavelet family. To this end, the normalized, complex representations of the time-by-frequency matrices for all sensors were subtracted from a reference electrode. Next, these complex difference values were averaged across trials, according to the algorithm described in [54,55]. This procedure results in a measure of phase locking between each electrode site and a reference site, across trials, for each time point and frequency, referred to as the phase-locking statistic (PLS) or inter-site phase locking [54]. The PLS is bounded between 0 and 1. A PLS of 0 indicates random distribution of the normalized phase difference between sensors across trials and 1 indicates perfect stability of the phase difference between two sensors across trials, at a given time and frequency. The reference electrode was FCz for gamma oscillations, and Cz for alpha oscillations, based on where power was maximal. Frontocentral sites FCz and Cz have been shown to capture electrocortical activity related to syllable and complex tone processing in a body of studies using event-related potentials, as well as indices of oscillatory activity [41,56,57,58]. They are therefore well suited as a reference point for analyses of large-scale cross-trial synchrony across locations. In addition, systematic laterality patterns of oscillatory activity with central topographical maxima have been observed previously in response to syllable learning [41]. Thus quantifying lateralized syllable processing with respect to a global and strong central response measured medially was expected to be sensitive to the hypothesized effects. Different reference locations were chosen for alpha and gamma because the stimulus-related changes in the alpha and gamma band (1) follow different time courses, (2) have different topography, and (3) show different direction of change over the baseline level. Note that our goal was not to draw direct comparisons between alpha- and gamma-band PLS, but to investigate changes in PLS in the two frequency band as a function of participant group and conditions. Using a reference electrode instead of the full matrix of PLS for each sensor pair helps addressing the accumulation of alpha error, and also ensures that the reference signal has a satisfactory signal-to-noise ratio. Similar procedures have been suggested, e.g., [59,60]. As discussed in these publications, scalp-based measures of coherency and phase synchrony are sensitive to volume conduction. The interpretation of absolute connectivity maps based on EEG is therefore not recommended. Source estimation has often been suggested as a preferable approach, but was not available in the present study, given the relatively sparse electrode montage. It has been argued, however, that comparing multiple experimental conditions, in which volume conduction can be considered stable, decreases the risk of spurious connectivity results [59,61]. PLS values are obtained as the power-normalized indices of phase stability, by averaging differences of complex phase values on a unit circle. Thus, in the absence of confounding factors (e.g., systematic differences in signal-to-noise between different conditions), reliable PLS differences between conditions are likely to reflect differences in the underlying neural synchrony, rather than the stable properties of the volume conductor. Caution is still warranted when interpreting connectivity based on scalp topographies, as mediating deep sources or additional processes may be undetected in scalp voltage data. Thus, focusing on one reference point for phase synchrony analysis may represent a limitation, but is also a conservative and careful first step, which relies on computationally manageable amounts of data, using a strong signal as the basis of the analysis.

The time-varying spectral power changes and time-varying PLS were extracted from two time-frequency windows relative to stimulus onset, one representing early (100–500 ms) oscillations in the alpha-band range (8–13 Hz), and one representing mid-range (200–400 ms) gamma-band activity (28–50 Hz). The selection of these time-frequency windows was based on grand mean time-frequency representations (see Supplementary materials, Figure 1). The goal of this selection was to obtain dependent variables with sufficient signal-to-noise ratio, which correspond to previous work. Both criteria are met in the present study. Specifically, alpha modulation starting early after stimulus presentation and being sustained for extended periods of time has been repeatedly reported in experiments with cross-modal or memory aspects [43]; likewise, auditory induced gamma in the 40 Hz range is a prominent and robust response to syllable stimuli [41]. The resulting spectral power and PLS for the alpha and gamma band ranges were evaluated using a repeated measures analysis of variance (ANOVA). Mean spectral power was analyzed separately for midline and lateral electrodes; PLS was analyzed at lateral sites only, since the PLS values were computed relative to a midline electrode. A separate analysis for the midline sites for the PLS would therefore involve fewer sites than the power analysis and would therefore not be comparable. Electrode regions were Fz, FCz, Cz, CPz and Pz for the midline analyses. Regions for the lateral analyses were: frontal (F3/4, F5/6, F7/8), fronto-central (FC3/4, FC5/6, FT7/8), central (left: C3/4, C5/6, T7/8), central-parietal (CP3/4,CP5/6, TP7/8), and parietal (P3/4,P5/6, P7/8). As in the ERP study on which the present study is based [12], we only analyzed data for low-falling and high-rising tones. We conducted two main analyses. In the first, we compared the pre-training data for all three groups; in the second, we compared the English and Chinese groups before and after training. Within-subject factors were tone (low-falling/high-rising), condition (standard/deviant), anteriority (levels: frontal, fronto-central, central, centro-parietal and parietal), and, where applicable, hemisphere (left/right), and test time (before/after training). Language group (3 or 2 levels depending on the analysis) was included as a between-subjects factor. The Greenhouse-Geisser correction was applied for effects involving factors with more than two levels, to control for sphericity violations [62]. The complete results of the ANOVAs are reported in the Supplementary materials. Significant interactions involving language group or location were followed-up with separate ANOVAs for each group or location.

## 3. Results and Discussion

### 3.1. Differences between the Groups before Training

**Figure 1:**
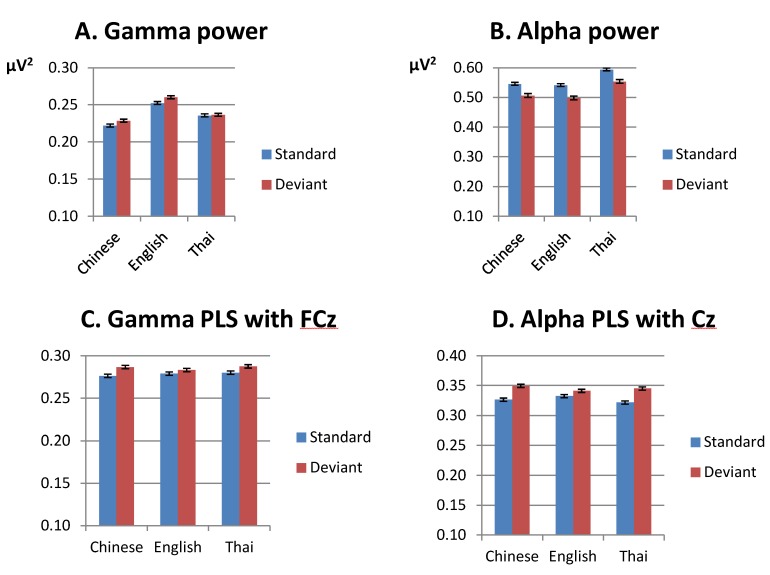
Means over all lateral electrodes for the three language groups before training, collapsed over tone. (**A**) gamma-band power, 200–400 ms after stimulus onset; (**B**) alpha-band power, 100–500 ms after stimulus onset; (**C**) gamma-band phase synchrony (PLS) with FCz, 200–400 ms after stimulus onset; (**D**) alpha-band phase synchrony (PLS) with Cz, 100–500 ms after stimulus onset. Note that the *y*-axis starts at 0.1, and that different scales are used for the different subfigures. The error bars represent the Standard Error.

Our first question concerned whether the native English, Chinese and Thai groups differed in the processing of the Thai tone stimuli before training, as reflected by differences in gamma-band power, alpha-band power, gamma-band phase synchrony and alpha-band phase synchrony. Figure 1 gives an overview of the differences between the three language groups before training. 

#### 3.1.1. Gamma Band, Spectral Power (200–400 ms from Stimulus Onset)

**Figure 2:**
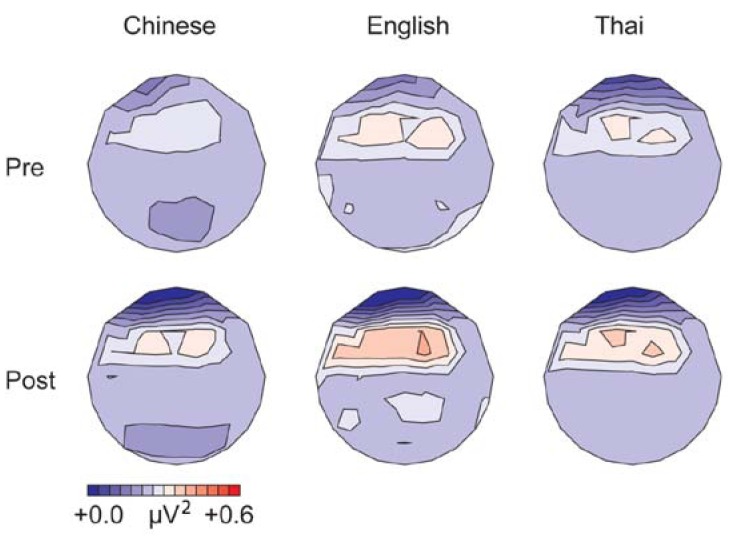
Mean spectral power in the gamma band (200–400 ms) for the three language groups, before (*pre*, upper row) and after training (*post*, bottom row), collapsed over tone and condition.

The upper row in Figure 2 displays the mean gamma band power for the three language groups before training, collapsed over tone and condition. Means for lateral electrodes are given in Figure 1A. Spectral power in the gamma band was largest over left frontal-central sites. Gamma-band power was stronger in the native English participants compared to the Thai and Chinese groups, and for the Thai compared to the Chinese group (effect of language: lateral sites, *F*(1,28) =68.31, *p* < 0.001; midline sites, *F*(1,28) = 7.66, *p* < 0.001). *Post-hoc* comparisons showed that all groups differed significantly from each other (*p* < 0.001 at lateral sites; *p* < 0.05 at midline sites, Bonferroni corrected).

#### 3.1.2. Alpha Band, Spectral Power (100–500 ms from Stimulus Onset)

**Figure 3:**
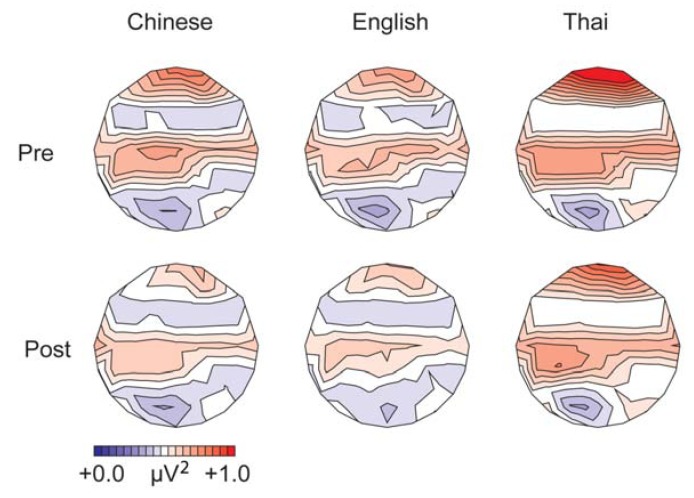
Mean spectral power in the alpha band (100–500 ms from stimulus onset) for the three language groups, before (*pre*, upper row) and after training (*post*, bottom row), collapsed over tone and condition. The large values at the extreme frontal sites are an artifact of the interpolation.

The upper row of Figure 3 displays the results for the alpha-band power before training. Means for lateral electrodes are given in Figure 1B. Alpha-band power before training was strongest over central and left centro-parietal lateral sites. Alpha-band power was stronger for standard than deviant trials (lateral sites, *F*(1,28) = 68.37, *p* < 0.001; midline sites, *F*(1,28) = 32.55, *p* < 0.001), replicating previous studies using oddball-paradigms, e.g., [63,64]. The language groups differed significantly (lateral, *F*(1,28) = 64.55, *p* < 0.001; midline, *F*(1,28) = 8.37, *p* < 0.001). The Thai speakers showed a stronger alpha-band power than the English and Chinese groups (*p* < 0.05 over midline sites; *p* < 0.001 over lateral sites), whereas the latter groups did not differ before training overall. The groups differed as to the effect of condition and tone, leading to a three-way interaction between tone, condition and language at midline sites (*F*(1,28) = 4.00, *p* < 0.05). Follow-up comparisons showed that the difference in alpha-band power between standard and deviant trials was larger for the low-falling than high-rising tones in the English speakers (*F*(1,9) = 6.20, *p* < 0.05), but did not differ significantly for the two tones in the Thai and Chinese participants (*p* > 0.17), see Table 1. This result patterns with the findings from the ERP analysis [12], in which the English speakers showed a larger mismatch negativity compared with the other groups for the low-falling deviant *versus* standard tones before training.

**Table 1:** Mean power in the alpha band (100–500 ms) for the low-falling and high-rising conditions for the deviants and standard stimuli for the three language groups, pre-and post training (standard error in parentheses).

Test time	Language group	Low-falling tones		High-rising tones	
		Standard	Deviant	Standard	Deviant
Pre training	Chinese	0.571 (0.021)	0.526 (0.023)	0.631 (0.018)	0.520 (0.019)
	English	0.570 (0.021)	0.477 (0.023)	0.580 (0.018)	0.566 (0.019)
	Thai	0.640 (0.020)	0.574 (0.021)	0.618 (0.017)	0.588 (0.018)
Post training	Chinese	0.567 (0.023)	0.465 (0.028)	0.582 (0.027)	0.507 (0.020)
	English	0.527 (0.023)	0.478 (0.028)	0.579 (0.027)	0.504 (0.020)

#### 3.1.3. Gamma Band, Phase Synchrony with FCz (200–400 ms from Stimulus Onset)

Analyses of phase synchrony (PLS) were conducted using lateral sites only (see Section 2). Means for lateral electrodes are given in Figure 1C. Gamma-band phase synchrony was calculated relative to FCz, and was strongest over the left fronto-central region. Gamma-band phase synchrony was stronger for deviant than standard trials (*F*(1,28) = 17.89, *p* < 0.001). No differences were found between the groups.

#### 3.1.4. Alpha Band, Phase Synchrony with Cz (100–500 ms from Stimulus Onset)

Means for lateral electrodes are given in Figure 1D. Phase synchrony with Cz was stronger for deviant trials than for standards (*F*(1,28) = 34.37, *p* < 0.001), especially at right central sites. The language groups differed in where the alpha-band phase synchrony between standards and deviants was maximal. In both the English and Chinese participants, the difference was maximal over the right hemisphere, whereas it was symmetric over the hemispheres for the Thai, leading to a four-way interaction of condition, anteriority, hemisphere and language (*F*(8,112) = 2.20, *p* < 0.05). In addition, the language groups differed in how the effect of condition was distributed for the two tones (*F*(8,112) = 2.15, *p* < 0.05): whereas the difference in phase synchrony between standard and deviant was largest over central sites for the low-falling tones in all groups, the largest difference between deviants and standards for the high-rising tones was reached more fronto-centrally for the Thai group compared to the other two groups. 

### 3.2. Differences in the Effects of Training between the Native English and the Native Chinese Language Groups

**Figure 4:**
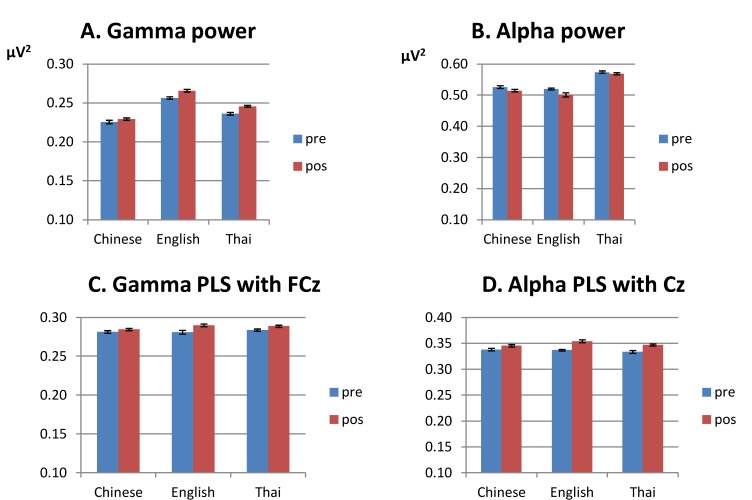
Mean for lateral electrodes for the three language groups, before and after training. (**A**) gamma-band power, 200–400 ms after stimulus onset; (**B**) alpha-band power, 100–500 ms after stimulus onset; (**C**) gamma-band phase synchrony (PLS) with FCz, 200–400 ms after stimulus onset; (**D**) alpha-band phase synchrony (PLS) with Cz 100–500 ms after stimulus onset. Note that the *y*-axis starts at 0.1 and that different scales are used for the different subfigures. Error bars are standard errors.

The second focus of the current analysis was to compare the effect of training in the native English and native Chinese groups. Figure 4 gives an overview of the differences between the language groups after *versus* before training in gamma-band power, alpha-band power, gamma-band phase synchrony and alpha-band phase synchrony. To assess the effect of training, we conducted an ANOVA on the pre- and post training data for the English and Chinese groups only. We will focus on effects involving the factor test time (pre-training, post-training) only. For completeness, data for the native Thai group are provided as well. We will discuss the outcomes for the Thai group in Section 3.2.6 below.

#### 3.2.1. Gamma-Band Power (200–400 ms from Stimulus Onset)

Pre and post-training data for the gamma-band power are displayed in the lower row in Figure 2. Means collapsed over the lateral electrodes are given in Figure 4A. We will first consider the native English and native Chinese group only. Gamma-band power increased after training (lateral sites, *F*(1,18) = 17.52, *p* < 0.001; midline, *F*(1,18) = 19.25, *p* < 0.001), especially at fronto-central sites (test time by anteriority, lateral, *F*(4,72) = 6.95, *p* < 0.001; midline, *F*(4,72) = 5.94, *p* < 0.05). The English group showed a larger increase in gamma-band power after training compared with the Chinese group at midline sites (test time by language, *F*(1,18) = 6.98, *p* < 0.05). Analyses for the groups separately showed a significant effect of training for the English group only (midline sites: *F*(1,9) = 18.84, *p* < 0.01).

#### 3.2.2. Alpha-Band Power (100–500 ms from Stimulus Onset)

Means alpha-band power for the pre and post training session is displayed for lateral sites in Figure 4B; post training data are displayed in the lower row of Figure 3. Overall, alpha-band power decreased after training compared with before (lateral sites, *F*(1,18) = 13.43, *p* < 0.001; midline, *F*(1,18) = 10.30, *p* < 0.001), especially at central and central-parietal sites, where alpha-band power was strongest before training (test time by anteriority, lateral, *F*(4,72) = 7.61, *p* < 0.01; midline, *F*(4,72) = 3.22, *p* < 0.05). No overall difference was found in the effect of training between the English and Chinese groups. However, patterns differed depending on condition and tone type, leading to a four-way interaction between test time, tone, condition and language at midline sites (*F*(1,18) = 5.31, *p* < 0.05). This was mainly due to the aforementioned finding that the English group showed a larger difference between standards and deviants for the low-falling than for the high-rising tones pre-training, which reduced post-training, see Table 2 (English: test time by condition by tone, *F*(1,18) = 7.28, *p* < 0.05; tone by condition pre-training, *F*(1,9) = 6.20, *p* < 0.05; post-training, *F*(1,9) = 0.19, *N.S.*).

#### 3.2.3. Correlation between Gamma and Alpha-Band Power

Hamame *et al.* [42] reported a strong negative correlation between gamma and alpha-band power in their training study. We therefore investigated whether there was a correlation between these two frequency bands for the Chinese and English groups in our study. We focused our analysis on locations where the gamma and alpha-band activation was largest, namely at left-fronto-central areas (FC3, FC5, FT7) and left central-parietal areas (CP3, CP5, TP7), respectively. Collapsed over collapsed over tone, condition, test time, and language group, we observed a significant negative correlation between the two frequency bands (Spearman’s ρ = −0.63, *p* < 0.01). 

#### 3.2.4. Gamma-Band, Phase Synchrony with FCz (200–400 ms from Stimulus Onset)

Pre- and post-training means for the gamma-band phase synchrony are given in Figure 4C for lateral electrodes. Phase synchrony in the gamma band was stronger after training compared with before (*F*(1,18) = 12.92, *p* < 0.001), especially over left central-frontal sites (test time by anteriority by hemisphere, *F*(4,72) = 3.21, *p* < 0.05). No differences were found between the language groups. 

#### 3.2.5. Alpha-Band, Phase Synchrony with Cz (100-500 ms from Stimulus Onset)

Means for lateral electrodes are given in Figure 4D. Collapsed over the Chinese and English groups, the alpha-band phase synchrony with Cz increased after training (*F*(1,18) = 47.28, *p* < 0.001), especially over central lateral sites, where alpha-band phase synchrony was maximal before training (test time by anteriority, *F*(4,72) = 7.65, *p* < 0.01). Note that this is in contrast to the pattern observed for the alpha-band power, which *decreased* after training. Training had a different effect on the groups, however, (test time by language, *F*(1,18) = 7.86, *p* < 0.01). The English group showed a stronger phase synchrony after training compared with the Chinese group (post training: English *vs*. Chinese: *F*(1,18) = 6.19, *p* < 0.05). The groups did not differ before training (*F*(1,18) = 0.17, *N.S.*). 

The pre-training right-lateralization of the difference between the standard and deviants reported above for the English and Chinese groups disappeared after training, leading to a four-way interaction of condition by hemisphere by anteriority by test time (*F*(4,72) = 3.80, *p* < 0.05).

#### 3.2.6. Effects of Training in the Thai Language Group

As mentioned in section 2, the native Thai group underwent the same type of training as the English and Chinese groups. We refrained from directly comparing the effect of training between the Thai and the other groups because the stimuli used were actual words in Thai, albeit edited speech. Since it is unclear what the effect is of training on familiar words, any differences in oscillatory brain activity post *versus* pre-training may therefore be hard to interpret. However, in order to give a complete picture of the data, we will report the effects of test time for this language group also. The data for this group are displayed in the right most columns of Figure 1, Figure 2, Figure 3 and Figure 4. A table of the outcomes of the statistical tests is provided in the supplementary materials. The native Thai group was similar to the English and Chinese groups in the following respects. First, gamma-band power increased after training at midline sites (*F*(1,10) = 6.34, *p* < 0.05). Here, the Thai group did not differ in the size of the training effect from the Chinese group (*F*(1,19) = 0.48, *p =* 0.49), but showed a smaller effect of training than the English (*F*(1,19) = 4.51, *p* < 0.05). Second, the Thai group showed an increase in alpha-band phase synchrony after training (*F*(1,10) = 24.99, *p* < 0.001), which we also observed in the Chinese and English groups. Direct comparisons between the three language groups of the difference between post- and pre-training alpha-band phase synchrony showed no significant effect of language group. Third, the Thai group showed shifts in the scalp distribution after training of the difference in gamma-band power, alpha-band power and alpha-band phase synchrony between standard and deviant conditions. More interestingly, and in contrast to what was observed for the English and the Chinese groups, however, the Thai group showed no significant increase in gamma-band phase synchrony (*F*(1,10) = 4.02, *p* = 0.07), or decrease in alpha-band power (*p* > 0.2), after training compared with before. Furthermore, whereas the Chinese and English group showed a negative correlation between power in the gamma-band and alpha-band, the Thai group did not show a correlation between the two frequency bands (Spearman’s ρ = 0.26, *p* = 0.43).

### 3.3. Discussion

#### 3.3.1. Overview of Results

This study explored changes in power and phase synchrony of EEG frequency bands in relation to the learning of new speech categories, in particular, lexical tones. We were interested in (1) differences between language groups before training; and (2) differences in the effect of training between the native English and the native Chinese language groups. A summary of the main results is given in Table 2.

**Table 2:** Overview of the main findings; *post* = post training; *pre* = pre training.

	Effect of language group before training (Thai, Chinese, English)	Effect of training (Chinese and English only)	Effect of training (Thai only)
*Gamma-band power*	English > Thai > Chinese	English: post > pre; Chinese: post = pre	Post > pre
*Alpha-band power*	Thai > English = Chinese English: difference between deviants and standards larger for low-falling than for high-rising tones	Post training < pre training English: pre-training difference between deviants and standards larger for low-falling than high-rising tones	Post = pre Differences in distribution of differences between standards and deviants before and after training
*Gamma-band phase synchrony*	English = Thai = Chinese	Post > pre English = Chinese	Differences in distribution of differences between standards and deviants before and after training
*Alpha-band phase synchrony*	Thai show symmetric distribution of differences between deviants and standards; English and Chinese show right hemisphere maximum	Post > pre pre: English = Chinese post: English > Chinese	Post > pre Differences in distribution of differences between standards and deviants before and after training

As for (1), the differences between language groups before training, the Thai, Chinese and English language groups differed before training in that the English showed the strongest gamma-band power, and the Chinese group the weakest. Alpha-band power, on the other hand, was strongest in the Thai group. Before training, the English group showed a larger difference between deviant and standards tones for the low-falling compared to the high-rising tones, whereas in the Thai and Chinese groups the difference between standards and deviants was not significantly modulated by the type of tone. The three groups did not differ in gamma-band phase synchrony. As for alpha-band phase synchrony, the native Thai group differed from the Chinese and English groups in the scalp distribution of the difference in synchrony between the standard and deviant trials. 

As for (2), the differences in the effect of training between the native English and the native Chinese language groups, the native English speakers, who were not familiar with linguistic tones, showed a larger increase in gamma after training compared with the Chinese group, who were familiar with tones in their own language. Both Chinese and English groups showed a decrease in alpha-band power after training. Finally, phase synchrony in both the gamma and alpha band was stronger *after* training than before, with the English group showing a stronger phase synchrony in the alpha-band than the Chinese after training. Effects were different in the Thai group, in that that subject group did not show any main effect of training on alpha-band power. In addition, no correlation was seen in the Thai group between alpha-band and gamma-band power, whereas this negative correlation was significant in the other two groups. This suggests that learning of new categories involves the relation between the gamma and alpha frequency bands, rather than changes within the gamma band separately. Below we will elaborate on the language and training effects observed for the gamma-band power, alpha-band power and alpha-band phase synchrony.

#### 3.3.2. Gamma-Band Power

Previous studies reported an increase in gamma-band power as a function of learning [40,41]. Increases in gamma power have been associated with the formation of neural representations and formation of networks [28], and the mapping of stimuli onto representations in memory [65]. An increase in gamma-band activation has also been found for words *versus* pseudo-words, which is attributed to the activation of the semantic/lexical system, e.g., [37,38]. Several mechanisms may therefore underlie the differences found in gamma-band power between the three groups before training. The larger gamma-band activation found in the Thai before training compared with the Chinese participants may be due to the fact that the syllables used were real words in Thai. The use of actual Thai words may therefore have lead to semantic activation, and hence, a larger gamma activity in the Thai than the Chinese participants, for whom the syllables did not have a pre-established meaning.

The stronger gamma-band activity found in the English group before training, and the larger increase in gamma-band power after training, compared with the other groups, may be due to other mechanisms. A speculative interpretation is that the larger gamma power in the English participants compared with Chinese reflects the formation of representations of the foreign tone categories, combining perceptual features, such as pitch slope and turning point, which are not relevant in English. The Chinese speakers, on the other hand, are already familiar with tones in their native language, and the dimensions that are relevant to form representations of the new tone categories. The continued exposure to the stimuli, even during the pre-training session may have already induced these changes. The larger gamma band activation for the English group may therefore reflect the formation of new networks in the brain, representing the tone categories. When the perceptual dimensions are known, but need to be combined slightly differently, as in the Chinese speakers, no new networks need to be recruited. If it is indeed the case that pre-training exposure induces changes in category formation, gamma-band power is expected to increase for the English group over the course of the pre-training session. We currently lack the power to further test this. Note that also the Thai group showed an increase in gamma-band power after training. This suggests that category formation is not reflected by changes in gamma-band power alone. Crucially, the gamma-band power did not correlate with alpha-band power in the Thai group. This suggests that learning involves the relation between the activity in the two frequency bands. We will return to this below.

#### 3.3.3. Alpha-Band Power

The interpretation of alpha-band activity is controversial, e.g., [43,44]. One interpretation of alpha-band activity is that it reflects a cortical “idling” process. When, e.g., sensory areas are stimulated, idling is reduced, leading to a smaller or less coherent alpha-band activity over these areas [66]. A smaller decrease in alpha as a result of stimulation or task has been associated with more efficient processing [46]. Given the fact that the stimuli were real words with familiar tones to our Thai-speaking participants, the larger alpha-activity seen in the native Thai group compared with the other groups can be interpreted as a more efficient processing for the native speakers of the stimulus language.

An alternative interpretation is that an increase in alpha-band activity reflects active inhibition of areas that are not relevant to the task [43,67]. In the present experiment, participants watched a silent movie while the tones were presented. Since the stimuli were real words in Thai, the native Thai speakers might therefore have more strongly inhibited the visual task, and shifted their attention to the auditory stream, resulting in a stronger alpha power in the Thai speakers *versus* the Chinese and English groups. This account is somewhat problematic however. If the increase in alpha activity reflects the shift of attention away from the visual stimuli, one would expect such an attention shift to occur especially when deviant auditory stimuli were presented. In contrast to this expectation, we found that alpha-band power decreased rather than increased with the presentation of deviant *versus* standard auditory stimuli.

Regardless of the interpretation of the alpha-band activity, the alpha-band power correlated negatively with the gamma-band power in the Chinese and English group, but crucially, not for the Thai group. This relation between alpha and gamma band activity suggests that both networks reflect aspects of learning, presumably the formation of new categories. Although the Thai group showed a modulation of gamma power as a result of training, the absence of a correlation with alpha-band power suggest that different mechanisms were involved in this group. A correlation between the alpha- and gamma-band power was also reported in the training study by Hamame *et al.* [42]. In this study, gamma-band activity first increased and then decreased over the course of learning, whereas alpha-band activity decreased and later increased. This was taken to reflect two phases of learning: one in which the number and/or strength of the neural connections increase to form a new neural representation (increase in gamma-band activity, decrease in alpha-band activity); and a second phase in which coding becomes more efficient by restricting the representation to the strongest or most selective connections (decrease in gamma-band activation and an increase in alpha-band activity). A potential extension of the current study would be to train participants for multiple days. If the model proposed by Hamame *et al.* [42] is correct, one would expect gamma-band power to decrease and alpha-band power to increase over the course of training for the Chinese and English groups.

Another observation in our current study with respect to the alpha-band power was that the English group showed a larger difference between standards and deviants for the low-falling tones than for the high-rising tones pre-training; this difference disappeared after training. The Thai and Chinese groups did not show significant effects of tone type on the difference between standards and deviants. Recall that the current analysis is based on data collected in a previous study [12]. In the ERP analysis reported in that study [12], the English group showed a larger mismatch negativity (MMN) effect between deviants and standards for the low-falling tones before training. This MMN effect was ascribed to the presence of a larger difference between the height of the onset pitch between the standards and deviants in the low-falling than in the high-rising tone conditions, to which the English learners may have been more sensitive before training than the Thai and Chinese. After training, the English may have learned to rely less on pitch onset and more on pitch contour. The observation that difference in alpha-band power between low-falling standards and deviants is larger before training than after training in the English group suggests a relation between alpha-band power and the MMN. However, we are careful in drawing any strong conclusions here, since other studies have suggested a correspondence between theta-band oscillations and the MMN instead [68,69,70].

#### 3.3.4. Gamma and Alpha-Band Phase Synchrony

Phase synchrony has been regarded to be a measure of the extent and efficiency of the networks involved. Here, we compare the statistical relationship between synchrony in both bands, without implying cross-frequency coupling on a physiological level. The three language groups differed before training in the distribution of the difference in alpha-band phase synchrony between the standard and deviant trials. The Chinese and English groups pattern together in this respect *versus* the Thai group. This again, is not surprising since the Thai group was expected to deal with the stimuli differently, since for this group the stimuli could be perceived as real, meaningful words. 

We observed a *stronger* gamma-band and alpha-band phase synchrony with increased proficiency, that is, after training in the English and Chinese groups. Recall that the mean spectral power in the alpha band *decreased* after training, as opposed to the increase in alpha-band phase synchrony. A potential interpretation of this pattern is that after training a more coherent, but smaller neural network was engaged for tone processing as reflected by alpha-band activity; the network reflected by gamma-band activity became both more coherent (increase in phase synchrony) and encompassed more active units (increase in power) after training, at least, for the Chinese and Thai. The alpha-band phase synchrony became stronger after training for the English compared to the Chinese group. Along the lines of Reiterer *et al.* [49], our data suggest that the poorer performers (the English participants in our case), recruit a more coherent network in response to training than more proficient listeners (Chinese in our case). However, we need to be cautious in this interpretation as also the Thai participants showed a stronger alpha-band synchrony after training. From a methodological perspective, caution is also warranted when using scalp-derived measures of connectivity because they are heavily influenced by volume conduction. In the present study, we adopted the strategy of comparing the PLS across conditions with similar signal-to-noise ratios, rather than making absolute claims about connectivity maps to address this issue. This strategy has been recommended [61]. Thus, although relative differences in PLS among the groups and conditions can be interpreted as reflective of brain electric activity, an in-depth analysis of cortical connectivity during syllable perception will likely involve measures such as functional imaging or intracranial measurements. Another potential cause for correlated power and synchrony changes among nearby electrodes is that either a deep or tangential source, or a large group of distributed neurons, project to the sensor group showing correlated power and synchrony changes (see [71], for simulations of how this affects EEG power topographies).Another potential drawback of our methods used is the use of locations with high signal-to-noise as seed regions in order to reduce spurious connectivity estimates. This method may result in neglecting potentially interesting connectivity information among sensors that are not part of the seed-related network. 

In contrast to the previous studies on language proficiency [48,49], we did not observe an overall hemispheric difference in coherence between the learning groups. Instead, we observed a right-hemispheric maximum for the difference between standards and deviants for both Chinese and English participants, which was reduced after training, suggesting that training affects lateralization of some aspects of processing. Note, however, that in the previous studies cited [48,49], EEG oscillations were analyzed over the entire course of the stimulus presentation, not specifically tied to particular stimuli, as in the current study. The partial correspondence between our findings and the previous studies therefore should be interpreted with caution.

## 4. Conclusions

The aim of this study was to investigate the effect of language background and short term training on the brain oscillations to lexical tone processing. We found an increase in gamma-band power and an increase in scalp-measured phase synchrony in the alpha-band after training for the native English group. These effects were smaller in the Chinese speakers in our study, who were already familiar with the use of tones in their own language. In addition, both Chinese and English groups, but crucially, not the native Thai participant group showed a negative correlation between alpha-band and gamma-band power. A tentative explanation of this is that the relation between gamma and alpha-band activity reflects the formation of new categories. Recombining existing features to form a category (as in the Chinese group) recruits fewer neural resources than combining new features (as in the English group). 

Time-frequency analysis therefore revealed effects of language background and training, which could not be observed in the previously reported ERP analysis [12]. Conducting both ERPs and time-frequency analysis therefore provides a more comprehensive view of speech processing and the effects of long- and short term exposure thereon. 

## Supplementary Files

Supplementary File 1 Supplementary Information (PDF, 626 KB)
