# Supplementary material for: Changes in Oscillatory Brain Networks after Lexical Tone Training
Source: Brain Sci. 2013 May 3;3(2):757–80. doi: 10.3390/brainsci3020757 (PMC4061855; doi:10.3390/brainsci3020757)
Supplement: Supplementary File 1 — Supplementary Information (PDF, 626 KB) [file brainsci-03-00757-s001.pdf]

Supplementary Information

Figure S1. Grand mean baseline-corrected evolutionary spectrum, collapsed over participant groups and test time, showing the time-varying spectral power for the deviant low-falling condition at electrode Cz (top) and FCz (bottom).

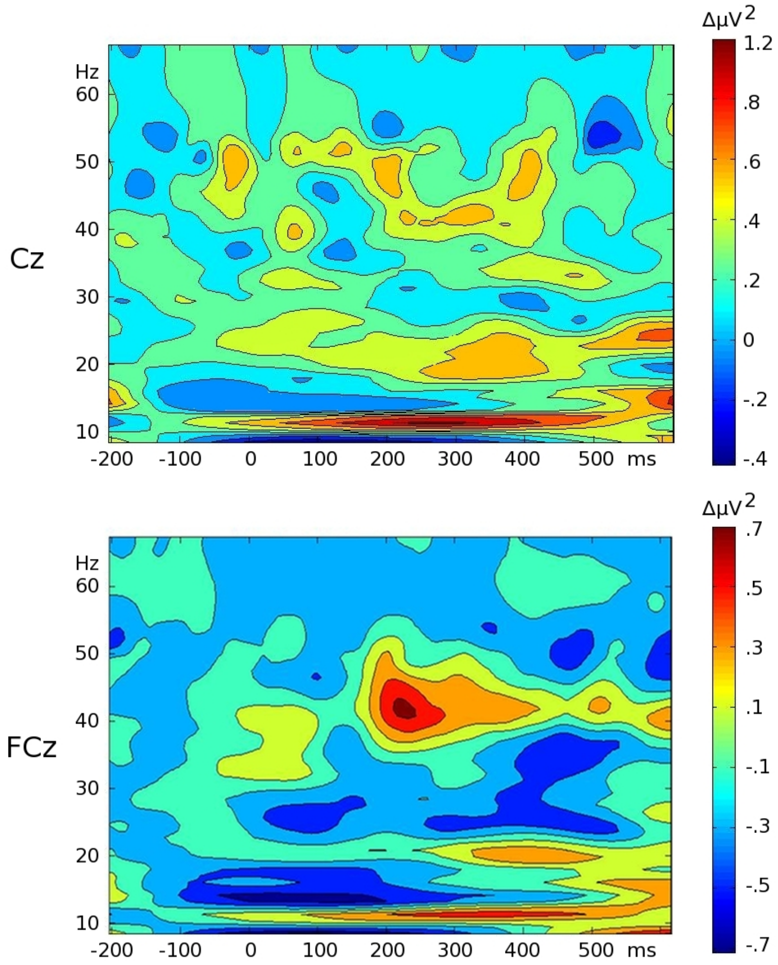

Table S1. Results from the ANOVAs on the pre-training data (comparing Thai, English and Chinese groups) on gamma and alpha-band power; analyses involving lateral and midline sites. DF and F are given. ** $p < 0.001$, * $p < 0.01$; $^{+}$ $p < 0.05$. *Ant* anteriority; *Cond* condition; *Hemi* hemisphere; *Lang* language group.

		Gamma-band power		Alpha-band power	
**Effect**	**DF**	**Lateral F**	**Midline F**	**Lateral F**	**Midline F**
Lang	1, 28	68.31 **	7.66 *	64.55**	8.37 **
Tone	1, 28	6.89 $^{+}$	5.88 $^{+}$	1.89	3.37
Tone × Lang	2, 28	0.61	0.08	2.19	1.41
Cond	1, 28	3.89	0.64	68.37 **	32.53 **
Cond × Lang	2, 28	0.75	1.24	0.05	0.78
Ant	4, 112	36.37 **	29.21 **	378.57 **	110.56 **
Ant × Lang	8, 112	0.86	0.71	1.69	1.48
Hemi	1, 28	1.32		26.13 **	
Hemi × Lang	2, 28	1.98		0.81	
Tone × Cond	1, 28	1.33	1.12	1.19	0.64
Tone × Cond × Lang	2, 28	0.72	0.82	0.15	4.00 $^{+}$
Tone × Ant	4, 112	2.65 $^{+}$	7.28 **	0.44	1.30
Tone × Ant × Lang	8, 112	0.98	0.53	1.83	0.50
Tone × Hemi	1, 28	0.71		0.03	
Tone × Hemi × Lang	2, 28	0.37		1.10	
Cond × Ant	4, 112	1.44	1.59	40.26 **	11.99 **
Cond × Ant × Lang	8, 112	0.23	0.11	0.86	0.60
Cond × Hemi	1, 28	0.97		4.53 $^{+}$	
Cond × Hemi × Lang	2, 28	2.70		1.61	
Ant × Hemi	4, 112	2.74 $^{+}$		56.54 **	
Ant × Hemi × Lang	8, 112	0.87		1.13	
Tone × Cond × Ant	4, 112	1.91	0.37	0.36	0.26
Tone × Cond × Ant × Lang	8, 112	1.73	0.33	0.41	1.36
Tone × Cond × Hemi	1, 28	0.43		0.34	
Tone × Cond × Hemi × Lang	2, 28	1.41		0.48	
Tone × Ant × Hemi	4, 112	1.62		1.63	
Tone × Ant × Hemi × Lang	8, 112	1.10		1.06	
Cond × Ant × Hemi	4, 112	0.17		14.03 **	
Cond × Ant × Hemi × Lang	8, 112	0.91		1.79	
Tone × Cond × Ant × Hemi	4, 112	0.09		0.76	
Tone × Con × Ant × Hemi × Lang	8, 112	0.63		0.19	

Table S2. Significant results from the ANOVAs on the pre-training data (comparing Thai, English and Chinese groups) on gamma and alpha-band PLS for analysis involving lateral sites. DF and F are given. ** $p < 0.001$, $^{+}$ $p < 0.05$. *Ant* anteriority; *Cond* condition; *Hemi* hemisphere; *Lang* language group.

		Gamma-band	Alpha-band
**Effect**	**DF**	**F**	**F**
Lang	1, 28	0.66	1.03
Tone	1, 28	3.47	1.81
Tone × Lang	2, 28	0.54	0.49
Cond	1, 28	17.89 **	34.37 **
Cond × Lang	2, 28	1.07	2.32
Ant	4, 112	116.36 **	169.56 **
Ant × Lang	8,112	0.95	0.48
Hemi	1, 28	216.54 **	0.00
Hemi × Lang	2, 28	0.31	0.62
Tone × Cond	1, 28	0.01	0.01
Tone × Cond × Lang	2, 28	2.72	1.11
Tone × Ant	4, 112	1.84	2.24
Tone × Ant × Lang	8, 112	0.64	1.57
Tone × Hemi	1, 28	2.17	0.02
Tone × Hemi × Lang	2, 28	0.11	0.59
Cond × Ant	4, 112	10.20 **	8.66 **
Cond × Ant × Lang	8, 112	1.02	0.67
Cond × Hemi	1, 28	18.40 **	6.03 $^{+}$
Cond × Hemi × Lang	2, 28	0.48	0.74
Ant × Hemi	4, 112	106.20 **	0.41
Ant × Hemi × Lang	8, 112	1.24	0.54
Tone × Cond × Ant	4, 112	0.35	0.60
Tone × Cond × Ant × Lang	8, 112	1.44	2.15 $^{+}$
Tone × Cond × Hemi	1, 28	1.16	3.10
Tone × Cond × Hemi × Lang	2, 28	0.00	0.91
Tone × Ant × Hemi	4, 112	2.78 $^{+}$	0.46
Tone × Ant × Hemi × Lang	8, 112	0.42	0.93
Cond × Ant × Hemi	4, 112	7.13 **	2.34
Cond × Ant × Hemi × Lang	8, 112	0.99	2.20 $^{+}$
Tone × Cond × Ant × Hemi	4, 112	0.39	1.42
Tone × Cond × Ant × Hemi × Lang	8, 112	1.49	0.97

Table S3. Significant effects in the ANOVAs on gamma and alpha-band power for pre- and post training data (English and Chinese language groups). Analyses involve lateral and midline sites. DF and F are given. ** $p < 0.001$, * $p < 0.01$; $^{+}$ $p < 0.05$. *Ant* anteriority; *Cond* condition; *Hemi* hemisphere; *Lang* language group.

		Gamma-band power		Alpha-band power	
**Effect**	**DF**	**Lateral F**	**Midline F**	**Lateral F**	**Midline F**
Lang	1, 18	291.47 **	81.10 **	3.80	0.90
Tone	1, 18	24.37 **	19.28 **	5.90 +	11.79 *
Tone × Lang	1, 18	0.75	1.03	0.28	0.61
Cond	1, 18	22.98 **	3.23	117.08 **	47.78 **
Cond × Lang	1, 18	1.70	2.13	0.88	1.60
Testtime	1, 18	17.52 *	19.25 **	13.43 *	10.30 *
Testtime × Lang	1, 18	3.10	6.98 +	0.80	0.10
Ant	4, 72	90.25 **	65.13 **	235.38 **	87.20
Ant × Lang	4, 72	2.26	3.22 +	1.36	1.50
Hemi	1, 18	6.67 +		64.94 **	
Hemi × Lang	1, 18	6.66 +		4.11	
Tone × Cond	1, 18	1.20	0.00	0.60	0.02
Tone × Cond × Lang	1, 18	0.61	3.36	0.02	0.81
Tone × Testtime	1, 18	0.43	0.19	1.05	0.02
Tone × Testtime × Language	1, 18	0.04	0.12	1.82	0.05
Tone × Ant	4, 72	3.16 +	8.57 **	3.46 +	3.93 +
Tone × Ant × Lang	4, 72	2.16	1.60	1.27	1.34
Tone × Hemi	1, 18	0.08		8.33 *	
Tone × Hemi × Lang	1, 18	0.05		2.95	
Cond × Testtime	1, 18	0.81	3.66	1.16	0.26
Cond × Testtime × Lang	1, 18	0.86	0.14	1.85	0.00
Cond × Ant	4, 72	2.14	2.15	68.96 **	17.17 **
Cond × Ant × Lang	4, 72	0.71	1.02	0.27	0.40
Cond × Hemi	1, 18	0.05		10.37 *	
Cond × Hemi × Lang	1, 18	4.73 +		0.12	
Testtime × Ant	4, 72	6.95 **	5.94 *	7.61 *	3.32 +
Testtime × Ant × Lang	4, 72	0.26	1.79	1.05	0.33
Testtime × Hemi	1, 18	4.27		0.60	
Testtime × Hemi × Lang	1, 18	0.43		0.01	
Ant × Hemi	4, 72	15.19 **		66.18 **	
Ant × Hemi × Lang	4, 72	2.18		1.48	
Tone × Cond × Testtime	1, 18	1.17	0.08	0.44	0.03
Tone × Cond × Testtime × Lang	1, 18	0.15	0.30	0.20	5.51 +
Tone × Cond × Ant	4, 72	1.55	0.397	0.21	0.25
Tone × Cond × Ant × Lang	4, 72	2.39	1.674	1.00	1.10
Tone × Cond × Hemi	1, 18	0.42		0.48	
Tone × Cond × Hemi × Lang	1, 18	1.57		0.07	
Tone × Testtime × Ant	4, 72	0.19	0.939	1.65	0.55
Tone × Testtime × Ant × Language	4, 72	0.62	0.451	1.25	0.51

Table S3. *Cont.*

Tone × Testtime × Hemi	1, 18	0.00		4.71 $^{+}$	
Tone × Testtime × Hemi × Lang	1, 18	0.11		0.25	
Tone × Ant × Hemi	4, 72	3.90 $^{+}$		5.96 *	
Tone × Ant × Hemi × Lang	4, 72	1.19		1.78	
Cond × Testtime × Ant	1, 18	0.27	0.031	1.43	0.67
Cond × Testtime × Ant × Lang	4, 72	0.59	1.34	0.79	0.66
Cond × Testtime × Hemi	1, 18	1.19		0.35	
Cond × Testtime × Hemi × Lang	1, 18	2.56		0.01	
Cond × Ant × Hemi	4, 72	1.07		20.90 **	
Cond × Ant × Hemi × Lang	4, 72	0.76		1.95	
Testtime × Ant × Hemi	4, 72	2.36		2.84	
Testtime × Ant × Hemi × Lang	4, 72	0.83		0.99	
Tone × Cond × Testtime × Ant	4, 72	2.69	1.39	1.29	0.66
Tone × Cond × Testtime × Ant × Lang	4, 72	0.76	0.80	0.86	1.77
Tone × Cond × Testtime × Hemi	1, 18	0.00		0.00	
Tone × Cond × Testtime × Hemi × Lang	1, 18	2.60		2.28	
Tone × Cond × Ant × Hemi	4, 72	0.47		0.65	
Tone × Cond × Ant × Hemi × Lang	4, 72	0.05		0.26	
Tone × Testtime × Ant × Hemi	4, 72	2.16		1.24	
Tone × Testtime × Ant × Hemi × Lang	4, 72	1.04		0.49	
Cond × Testtime × Ant × Hemi	4, 72	0.36		0.34	
Cond × Testtime × Ant × Hemi × Lang	4, 72	2.34		0.63	
Tone × Cond × Testtime × Ant × Hemi	4, 72	0.67		1.11	
Tone × Cond × Testtime × Ant × Hemi × Lang	4, 72	0.28		0.88	

Table S4. Significant results from the ANOVAs for pre- and post training data (English and Chinese language groups) on gamma and alpha-band PLS for analysis involving lateral sites. DF and F are given. ** $p < 0.001$, * $p < 0.01$; $^{+}$ $p < 0.05$. *Ant* anteriority; *Cond* condition; *Hemi* hemisphere; *Lang* language group.

		Gamma-band	Alpha-band
**Effect**	**DF**	**F**	**F**
Lang	1, 18	1.42	1.94
Tone	1, 18	7.01 $^{+}$	4.78 $^{+}$
Tone × Lang	1, 18	0.41	0.80
Cond	1, 18	17.58 *	22.59 **
Cond × Lang	1, 18	1.74	4.97 $^{+}$
Testtime	1, 18	12.92 *	47.28 **
Testtime × Lang	1, 18	2.89	7.86 $^{+}$
Ant	4, 72	236.62 **	395.41 **
Ant × Lang	4, 72	1.20	1.39
Hemi	1, 18	322.75 **	0.64
Hemi × Lang	1, 18	0.10	0.13
Tone × Cond	1, 18	0.01	2.56
Tone × Cond × Lang	1, 18	2.87	0.41
Tone × Testtime	1, 18	0.22	0.06
Tone × Testtime × Language	1, 18	0.02	3.70
Tone × Ant	4, 72	1.31	1.64
Tone × Ant × Lang	4, 72	0.14	0.52
Tone × Hemi	1, 18	2.02	1.45
Tone × Hemi × Lang	1, 18	1.15	0.40
Cond × Testtime	1, 18	0.02	0.04
Cond × Testtime × Lang	1, 18	0.18	0.00
Cond × Ant	4, 72	7.79 **	4.93 *
Cond × Ant × Lang	4, 72	1.42	0.65
Cond × Hemi	1, 18	13.55 *	4.57 $^{+}$
Cond × Hemi × Lang	1, 18	1.18	0.80
Testtime × Ant	4, 72	2.08	7.65 *
Testtime × Ant × Lang	4, 72	0.40	0.05
Testtime × Hemi	1, 18	2.41	0.01
Testtime × Hemi × Lang	1, 18	0.04	1.74
Ant × Hemi	4, 72	166.08 **	1.05
Ant × Hemi × Lang	4, 72	3.27 $^{+}$	0.81
Tone × Cond × Testtime	1, 18	0.83	0.11
Tone × Cond × Testtime × Lang	1, 18	2.83	0.52
Tone × Cond × Ant	4, 72	0.37	0.67
Tone × Cond × Ant × Lang	4, 72	0.48	0.51
Tone × Cond × Hemi	1, 18	0.03	2.61
Tone × Cond × Hemi × Lang	1, 18	0.01	12.36 *
Tone × Testtime × Ant	4, 72	1.15	1.22
Tone × Testtime × Ant × Language	4, 72	1.32	1.78
Tone × Testtime × Hemi	1, 18	0.33	0.85

Table S4. *Cont.*

Tone × Testtime × Hemi × Lang	1, 18	1.93	3.42
Tone × Ant × Hemi	4, 72	2.69	0.17
Tone × Ant × Hemi × Lang	4, 72	0.17	0.27
Cond × Testtime × Ant	1, 18	1.70	1.63
Cond × Testtime × Ant × Lang	4, 72	0.75	0.77
Cond × Testtime × Hemi	1, 18	0.98	3.56
Cond × Testtime × Hemi × Lang	1, 18	0.01	0.94
Cond × Ant × Hemi	4, 72	5.96 *	1.74
Cond × Ant × Hemi × Lang	4, 72	0.43	1.06
Testtime × Ant × Hemi	4, 72	3.21 $^{+}$	0.69
Testtime × Ant × Hemi × Lang	4, 72	0.39	0.72
Tone × Cond × Testtime × Ant	4, 72	0.33	1.95
Tone × Cond × Testtime × Ant × Lang	4, 72	0.35	0.65
Tone × Cond × Testtime × Hemi	1, 18	0.92	0.44
Tone × Cond × Testtime × Hemi × Lang	1, 18	0.00	0.69
Tone × Cond × Ant × Hemi	4, 72	1.63	0.67
Tone × Cond × Ant × Hemi × Lang	4, 72	1.61	1.38
Tone × Testtime × Ant × Hemi	4, 72	0.82	1.62
Tone × Testtime × Ant × Hemi × Lang	4, 72	0.85	0.45
Cond × Testtime × Ant × Hemi	4, 72	0.13	3.80 $^{+}$
Cond × Testtime × Ant × Hemi × Lang	4, 72	1.56	0.45
Tone × Cond × Testtime × Ant × Hemi	4, 72	0.28	0.46
Tone × Cond × Testtime × Ant × Hemi × Lang	4, 72	0.36	0.77

Table S5. Significant effects involving the factor Test time from the ANOVAs for the Thai group only, on gamma and alpha-band power and PLS. DF and F are given. ** $p < 0.001$, * $p < 0.01$; $^{+}$ $p < 0.05$. *Ant* anteriority; *Cond* condition; *Hemi* hemisphere.

**Measure**	**Analysis sites**	**Significant effects**
Gamma-band power	Midline	Test time, $F(1,10) = 6.34^{+}$
	Lateral	Test time, $F(1,10) = 14.94$ *; Test time by Ant, $F(4,10) = 4.07^{+}$; Test time by Cond by Tone by Hemi, $F(1,10) = 5.14^{+}$
Alpha-Band power	Lateral	Test time by Cond by Ant, $F(4,10) = 5.87$ *
Gamma-Band PLS	Lateral	Test time by Ant , $F(4,40) = 3.30^{+}$
Alpha-Band PLS	Lateral	Test time, $F(1,10) = 25.00$ **; Test time by Ant, $F(4,10) = 6.16$ *; Test time by Cond by Tone by Hemi, $F(4,40) = 3.64^{+}$

© 2013 by the authors; licensee MDPI, Basel, Switzerland. This article is an open access article distributed under the terms and conditions of the Creative Commons Attribution license (http://creativecommons.org/licenses/by/3.0/).
